# Effects of Matrix Modification on the Mechanical Properties of Wood–Polypropylene Composites

**DOI:** 10.3390/polym9120712

**Published:** 2017-12-14

**Authors:** Shunmin Yi, Shihua Xu, Yiqun Fang, Haigang Wang, Qingwen Wang

**Affiliations:** 1 Key Laboratory of Bio-Based Material Science and Technology (Ministry of Education), Northeast Forestry University, Harbin 150040, China; shunmyi@163.com (S.Y.); xush@nefu.edu.cn (S.X.); yqfang@nefu.edu.cn (Y.F.); 2 College of Materials and Energy, South China Agricultural University, Guangzhou 510642, China

**Keywords:** matrix modification, chemical graft, polymer–matrix composites, fiber–matrix interface, mechanical properties

## Abstract

Polypropylene (PP) modified with two reactive monomers, divinyl benzene (DVB) and maleic anhydride (MAH), was used as the matrix to prepare wood–polypropylene composites to improve interfacial compatibility. The effects of the co-modified PP matrices with different DVB concentrations on the mechanical properties of the composites were evaluated. Compared with unmodified composites and the composites containing a coupling agent, the composites modified with MAH only, and that with both MAH and DVB, improved the tensile, flexural, and impact strengths. Interestingly, adding a small amount of DVB (0.4%) resulted in significant increase in impact strength, relative to that of the composites modified with MAH only. Dynamic mechanical analysis and fracture morphology analysis of the modified composites also suggested an improvement in interfacial adhesion owing to the matrix modification.

## 1. Introduction

The use of thermoplastic polymers reinforced with wood fibers (WF) has received considerable attention owing to the low energy consumption and environmentally friendly nature of the materials [1,2,3]. Wood flour/plastic composites, which combine favorable properties with a low specific mass, have been widely used for non-structural applications, such as decking materials, automotive components, and packing products [4,5,6].

The hydrophilic property of WF, similar to all natural fibers, has an inherently low compatibility with hydrophobic polymers, such as polypropylene (PP) and polyethylene (PE) [7]. This incompatibility is unfavorable for interfacial fiber–matrix adhesion and dispersion of the fibers, resulting in inferior mechanical properties of the composites [8]. Various studies indicate that the fiber–matrix interaction plays a critical role in stress transfer between the matrix and the fibers, thereby affecting the mechanical properties of composites [9,10].

To improve the interfacial adhesion between WF and plastics, chemical or physical modification of WF or the matrix, and the addition of a coupling agent, can be used [11,12,13]. Adding a coupling agent is known to efficiently improve interfacial adhesion by forming a chemical bridge between the fibers and the matrix [14,15,16]. Maleated polypropylene (MAPP) is a well-known coupling agent for wood–plastic composites [17]. Aggarwal et al. reported that in jute–PP composites at 50% fiber concentration, the addition of 5% *m*-isopropenyl-α-α-dimethylbenzyl-isocyanate (m-TMI)-grafted-polypropylene as a coupling agent led to an increase in tensile and flexure strengths over virgin polypropylene, up to 87% and 95%, respectively [18]. Cabtero et al. reported that fiber modification with sodium hydroxide also improved the interfacial compatibility and properties of the composites [19]. Xie et al. demonstrated that treatment of WF with glutaraldehyde and 1,3-dimethylol-4,5-dihydroxyethyleneurea enhanced the interfacial compatibility and tensile strength of the composites [20].

Plastic matrix modification is another potential strategy to improve the mechanical properties of wood–polypropylene composites [21,22], which involves fewer solvent-based processes, in comparison with fiber modification. Effective chemical modification of polymer matrices has recently been developed via reactions with polar monomers to enhance interfacial adhesion, particularly in the case of polyolefin based composites. Gonzalez-Sanchez reported that for recycled low-density polyethylene matrix, adding peroxide in small amounts resulted in a remarkable improvement in the mechanical properties of the composites [23]. Grafting of glycidyl methacrylate onto a PP matrix improved the fiber dispersion in the PP matrix and enhanced the interfacial adhesion relative to that of unmodified composites [22]. Similar findings were reported for composites based on PP/PE blends grafted with maleic anhydride (MAH) [24]. With the addition of only 1 wt % MAH, the tensile, flexure, and impact strengths of PP/PE composites increased by 216%, 120%, and 62.7%, respectively, relative to those of the unmodified PP/PE composites. However, high initiator concentrations of peroxides are typically required to achieve moderate grafting efficiency, owing to the poor reactivity of MAH toward polypropylene macroradicals. Degradation of polymer matrix by chain scission occurs with increasing concentration of the initiator, which negatively affects the mechanical properties of PP-based composites.

Over the past decades, various strategies have been proposed to control PP degradation in melt free-radical grafting, by changing the radical intermediate states to stabilize PP macroradicals [25,26]. This strategy involves the use of comonomer styrene, *p*-(3-butenyl) styrene, or furan derivatives. Some reactive comonomers containing more polymerizable groups have also been used as crosslinking agents for polyolefin, owing to their high free-radical reactivity, for strength enhancement. Therefore, to confer superior mechanical properties of composites based on PP matrix modification, these monomers can be used to restrain the degree of PP degradation and to obtain moderate MAH grafting efficiency. This study is, thus far, the first to explore the effect of PP matrix modification on the mechanical properties of wood–polypropylene composites, where using divinyl benzene (DVB) as a second monomer in MAH-grafted PP matrices.

In this current study, DVB containing two polymerizable groups was used as an interlinking agent in PP–MAH grafting systems. The matrix with both MAH and DVB was modified with an extruder, and then compounded with WF to prepare the composites. The effects of co-modified PP matrices on the mechanical properties of composites were investigated. In addition, the fiber–matrix interface and the dispersion of wood fibers in the composites were characterized using scanning electron micrographs (SEM) and dynamic mechanical analysis (DMA).

## 2. Materials and Methods

### 2.1. Materials

Polypropylene (T30S) with a melt flow index of 3.8 g/10 min at 230 °C and 2.16 kg, in accordance with ASTM D1238-13, was supplied by Daqing Petrochemical Company in Daqing, China. Maleated polypropylene (with a melt flow index of 95 g/10 min at 230 °C and 2.16 kg, maleic anhydride grafting level of 1%) was used as a coupling agent (Shanghai Rizhisheng New Technology Development Co., Ltd., Shanghai, China). Maleic anhydride (MAH), dicumyl peroxides (DCP), divinyl benzene (DVB), and xylene were reagent-grade (Tianjin Bochen Co., Ltd., Tianjin, China). All chemicals were used as received. Poplar (*Populus alba*) wood veneers were ground into particles capable of going through a 40-mesh and being retained on a 80-mesh sieve, supplied by Harbin Yongxu Company, Harbin, China.

### 2.2. Sample Preparation

#### 2.2.1. Modification of Matrix

In the laboratory setting, grafting of MAH and DVB on PP (PP/MAH/DVB/DCP = 100/1/1/0.1 in weight) was conducted in a HAAKE torque rheometer (Thermo Fisher Scientific (China) Co., Ltd., Shanghai, China) at 180 °C and 60 rpm for 8 min. MAH, DCP, and DVB were first dissolved in acetone before being sprayed onto the granules of PP.

In the pilot-scale setting, grafting of MAH and DVB on the PP matrix was conducted in a co-rotating twin screw extruder (SJ65, Nanjing Rubber and Plastics Machinery Co., Ltd., Nanjing, China) at a rotary speed of 300 rpm and a reaction zone temperature of 180 °C with a total throughput of about 0.57 kg/min (Table 1). The barrel temperature profile from the hopper to the die was as follows: 140, 150, 170, 180, 190, 190, 190, 190, 190, 190, 200, 195, 180, and 170 °C.

#### 2.2.2. Preparation of Composites

The modified PP and wood fibers (WF) were dried in an oven at 103 °C for 24 h. WF, PP (virgin or modified), and MAPP were mixed at different ratios (Table 2) in a high-speed mixer for 10 min. The mixture was granulated with a twin-screw extruder (JSH30, Nanjing Rubber-Plastic Machine Ltd., Nanjing, China). A single-screw extruder (SJ45, Nanjing Rubber-Plastic Machine Ltd., Nanjing, China) was used to prepare WF/PP composite sheets. The processing temperatures of extrusion were: 145 °C in the melting zone, 155–180 °C in the pumping zone, and 177 °C in the die zone. The rotation speeds of the twin-screw and single-screw extruder were 50 and 20 rpm, respectively. 

### 2.3. Characterization

#### 2.3.1. Fourier-Transform Infrared (FTIR) Spectroscopy Analysis

The modified PP samples were dissolved in hot xylene at 140 °C for 1 h, and excess acetone was added to precipitate them. The precipitated samples were filtered, washed, and dried under vacuum at 100 °C for 24 h. Virgin PP (unmodified) as the control group, was also treated using the same procedure. Purified samples were pressed into films at 190 °C, and then analyzed using a Nicolet Magana-IR 560 spectrometer (Thermo Nicolet Corporation, Madison, WI, USA) at room temperature. Data were collected from 400 cm^−1^ to 4000 cm^−1^, with 32 scans for each sample. The resolution was 4 cm^−1^.

#### 2.3.2. Determination of Melt Flow Index and MAH Grafting Level of the Modified Matrix

The MAH grafting level relative to PP was tested by using a chemical titration method [27].

The melt flow index (MFI) was measured by using a Davenport melt flow indexer at 230 °C, with a weight of 2.16 kg in accordance with ASTM D-1238-13.

#### 2.3.3. Determination of Strength Properties

Both the PP matrices and the composites were prepared as dumbbell-shaped samples with the following dimensions: length, 165 mm; width at the end, 20 mm; width at the narrow portion, 12.7 mm; and thickness, 4 mm. Tensile tests were conducted in accordance with ASTM D638-10 at 50 mm/min for the PP matrix, and ASTM D638-10 at 5 mm/min for the composites. In accordance with ASTM D790-10, the samples with 80 mm × 12.7 mm × 4 mm were prepared for flexure tests. The test was carried out at 2 mm/min with a span of 64 mm. Both tensile and flexural tests were conducted with a RGT-20A universal testing machine (Shenzhen, China). Unnotched-Izod impact test was conducted with an impact tester (XJ-50G, Chengde, China) in accordance with ASTM D4812-06, with a sample size of 80 mm × 10 mm × 4 mm. All tests were performed 8 times at a relative humidity of 50% and a temperature of 23 °C.

#### 2.3.4. Dynamic Mechanical Analysis

All samples measured 35 mm × 12 mm × 2.8 mm, and were tested with a dynamic mechanical analyzer (2980 DMA V1.7B, TA, New Castle, DE, USA). The tests were conducted in the flexural mode with a frequency of 1 Hz over a temperature range of −50 to 125 °C at a rate of 5 °C/min.

#### 2.3.5. Morphological Analysis

The samples were frozen in liquid nitrogen for 5 min, and then broken down manually. The fractured surface was subsequently sputtered with gold, and then characterized with a scanning electron microscope (SEM, Quanta 200, FEI Company, Hillsboro, OR, USA) at an acceleration voltage of 12.5 kV.

## 3. Results and Discussion

### 3.1. Characterization of Chemically Grafted Polypropylene

**Figure 1:**
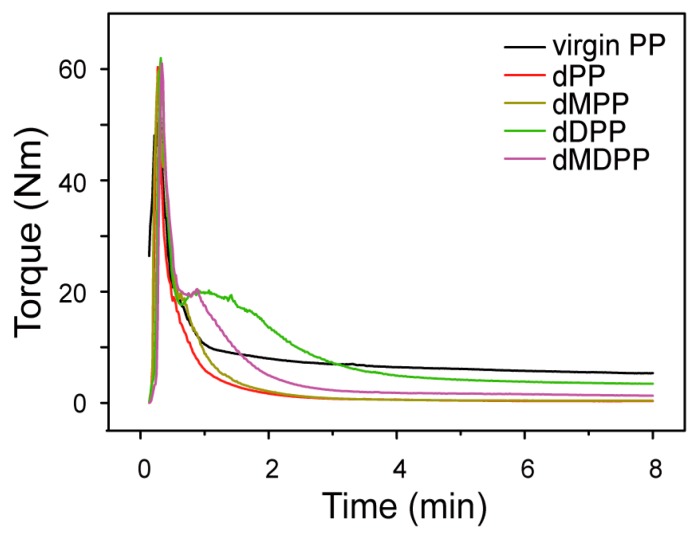
Torque behaviors of virgin PP, dPP (PP + DCP), dMPP, dDPP, and dMDPP systems ([MAH] = 1 wt %, [DCP] = 0.1 wt %, [DVB] = 1 wt %).

**Figure 2:**
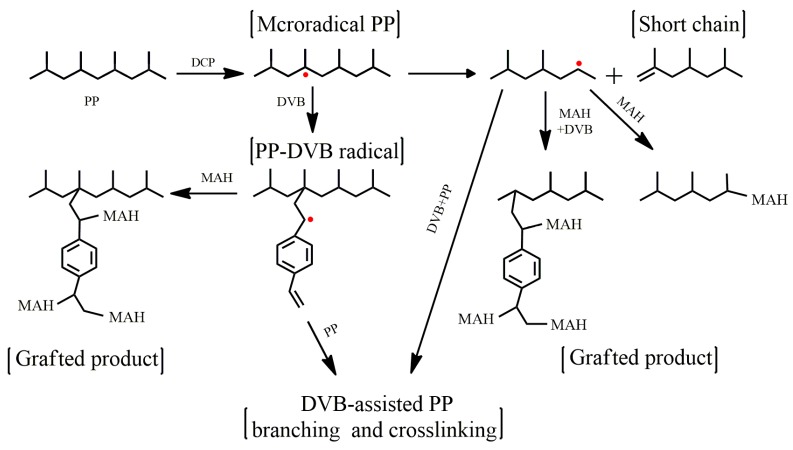
The main reactions related chemical grafting of MAH to PP in the presence of DVB.

The torque behavior of the peroxide (DCP)-initiated melt grafting system was examined with a HAAKE mixer (Figure 1). With the addition of DCP, the mixing torque values in the PP (dPP) and PP/MAH (dMPP) systems decreased gradually until the end of the reaction with an end-torque value lower than that of virgin PP. This reduction could be attributed to the severe chain scission of the PP backbone (via the tertiary macroalkyl radical) [28]. In the case of the system containing DVB (dDPP), a noticeable second torque peak was observed at the early stage, which also exhibited a lower end-torque value than that of virgin PP, but remained higher than those of dPP and dMPP. The reason was that the highly reactive DVB was preferentially grafted to PP macroradicals, and formed styryl macroradicals, thus restraining the β-chain scission reaction of PP macroradicals [28]. The grafted DVB further reacted with PP macroradicals and resulted in branching or crosslinking, which corresponds to the second torque peak [26,29]. After the DVB was consumed, the polymer degradation induced by residual peroxide could be dominated by β-chain scission reactions, which corresponds to a slow reduction in torque values at the later stage of the reaction. When both MAH and DVB were added (dMDPP), the peak intensity of the second mixing torque peak and end-torque values were significantly reduced, indicating that parts of the grafted DVB on PP macroradicals were reacted with MAH apart from DVB-assisted branching and crosslinking. The main grafting and competing reactions are shown in Figure 2.

**Figure 3:**
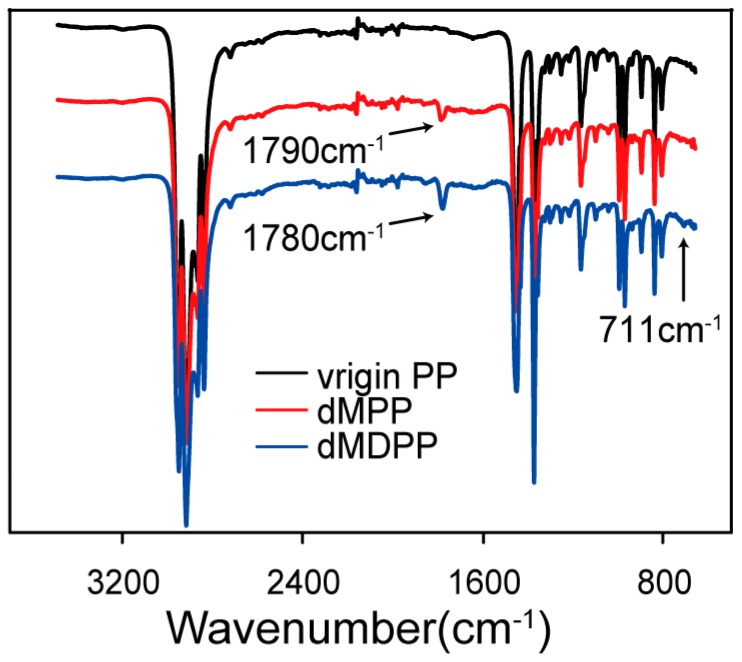
FTIR spectra of virgin PP, dMPP, and dMDPP ([MAH] = 1 wt %, [DCP] = 0.1 wt %, [DVB] = 1 wt %).

The FTIR spectra of unmodified PP and modified (dMPP, dMDPP) samples provided structural information of grafting monomers to PP backbone (Figure 3). New absorption bands at 1790 and 711 cm^−1^ were observed, which can be assigned to the carbonyl groups (–C=O) of the grafted MAH and the grafted DVB, respectively [25]. In comparison with the characteristic absorption of carbonyl groups in dMPP, the addition of DVB (dMDPP) shifted the characteristic absorption of carbonyl groups from 1790 cm^−1^ to 1780 cm^−1^. This occurrence may be attributed to the structural change in the grafted MAH, from a single succinic anhydride (grafted on the end chain of the PP backbone, with absorption at 1790 cm^−1^) to other forms of MAH, due to the reaction between DVB and MAH on the PP backbone, which corresponded to the aforementioned analysis of torque behavior [25].

**Figure 4:**
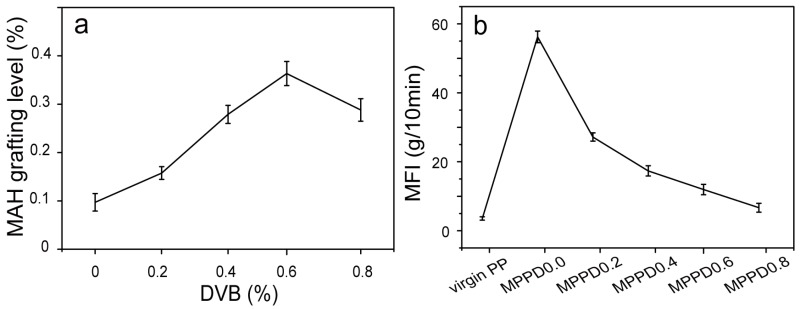
Effects of DVB concentration on the relative MAH grafting level (**a**) and MFI of PP matrix (**b**).

The modified PP matrix at the pilot-plant setting, in the presence of DVB, was also prepared using a twin-screw extruder. As illustrated in Figure 4a, the MAH grafting level increased with increasing DVB concentration, and then decreased. The results indicated that the high-reactivity comonomer DVB could act as an interlinking agent between the PP chain and MAH, resulting in an increase in the MAH grafting efficiency. With a further increase in DVB concentration (above 0.6%), predominance of DVB-assisted branching and crosslinking, rather than MAH grafting would occur, as indicated by a decrease in the MAH grafting level and the reduction in MFI (Figure 4b). The MFI of the modified PP decreased with increasing DVB concentration, but remained higher than that of the virgin PP. This finding suggested that not all tertiary macroradicals can be converted to styryl macroradicals by reacting with DVB to restrain chain scission reactions; the main grafting and branching reaction may occur after chain scission of PP [27,28].

**Figure 5:**
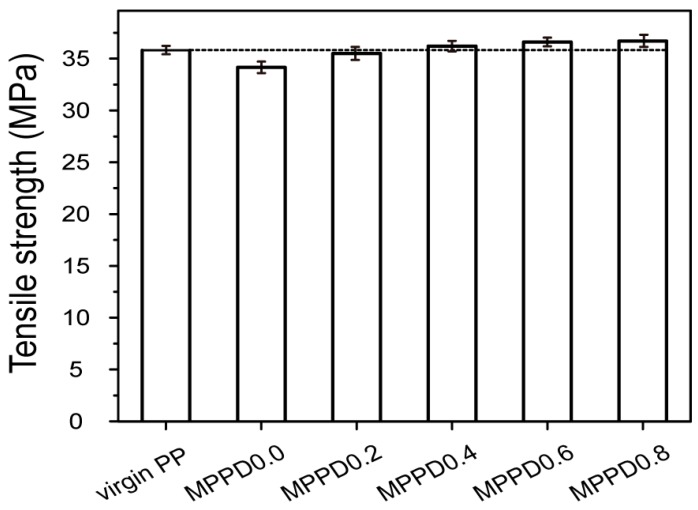
Tensile strength of virgin PP and the modified PP.

According to the theory of polymer strength, these molecular characteristics of the entanglement of branching and crosslinking structure can improve the mechanical properties of the modified PP [25]. Therefore, with the addition of DVB, the tensile strength of the modified PP slightly increased. When DVB concentration was above 0.2%, the tensile strength became even higher than that of the virgin PP (Figure 5). This improvement could be attributed to the DVB-assisted branching and crosslinking, which decreased the chain scission reactions, thus reducing the breakage of molecules and slippage between polymer chains [28].

### 3.2. Characteristics of the Composites

#### 3.2.1. Mechanical Properties of the Composites

According to the aforementioned results, the virgin PP exhibited higher tensile strength in comparison with MPPD0, whereas the composites (WMPD0) with MPPD0 as the matrix exhibited increases of 221%, 178%, and 75% in tensile, flexural, and impact strengths relative to the unmodified composites (WP) (Table 3). The increase could be attributed to the effective coupling by the covalent link between the anhydride carbonyl of the modified matrix and hydroxyl groups of wood surfaces, as well as the sufficient wettability of the matrix (MPPD0) with high MFI values, given that the applied force was more efficiently transferred from the matrix to WF. Compared with the modulus of the unmodified composites, the tensile and flexural moduli of MPPD0 were decreased to 8% and 11%, respectively. This result may be attributed to the PP matrix degradation via chain scission reactions. Comparison between the composites (WPMA) containing the MAPP coupling agent and WMPD0 was also studied. The results indicated that much less improvement in mechanical properties by matrix modification with MAH only was observed (Table 3), which could be explained by the following: (i) the lower MAH concentration resulted in a lower MAH grafting level, which failed to provide much more effective fiber–matrix coupling for stress transfer from the modified PP to WF [24]; and (ii) MPPD0 underwent chain scission, which were prone to achieving more effective wetting of WF by MPPD0, thereby promoting the formation of effective coupling between WF and the modified matrix; however, the inferior strength of MPPD0 itself with respect to virgin PP offset the positive effect from improved interfacial adhesion [24].

**Figure 6:**
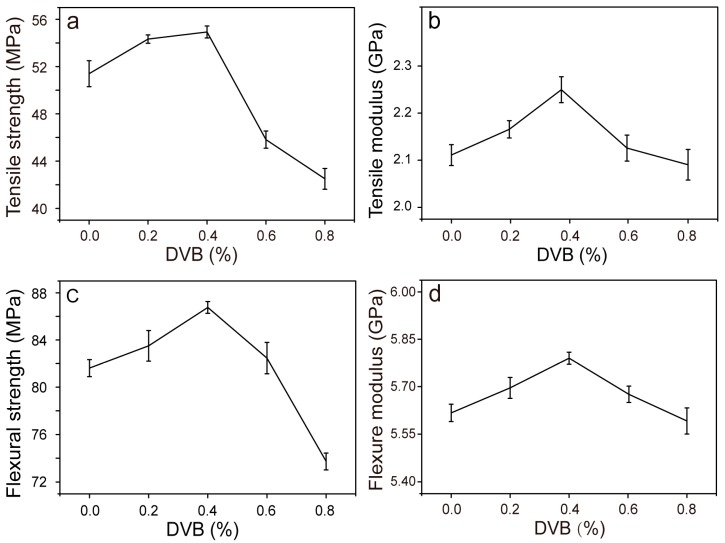
Mechanical properties of the composites modified with various DVB concentrations. (**a**) Tensile strength; (**b**) tensile modulus; (**c**) flexural strength; and (**d**) flexural modulus.

The mechanical properties of modified composites with different DVB concentrations were also evaluated (Figure 6). The tensile strength and modulus of the modified composites exhibited an increasing trend with respect to WMPD0 when the DVB concentration was increased to 0.4%. Above that value, the tensile strength and modulus decreased (Figure 6a). The increase in tensile strength could be attributed to the improved interfacial adhesion, owing to an increase in the MAH grafting level (Figure 4), as well as the improvement in the tensile strength of the matrix (Figure 5), which benefited the stress transfer from the matrix to WF. A further increase in initial DVB concentrations led to an increase in the grafting level of MAH; however, the modification process was also accompanied by a higher degree of branching and crosslinking, which could lead to insufficient wetting of WF, due to the matrix with lower MFI. The results indicate that beyond the critical concentration of DVB (0.4%) for the modified composites, in the case of constant MAH and DCP concentrations, the wetting of WF by the modified matrix with a lower melt flow rate may decrease, thus forming a weak effective coupling between the fibers and the matrix. The flexural strength and modulus of the composites reflected the same change trend as that of the aforementioned tensile properties by varying the DVB concentration (Figure 6c,d).

**Figure 7:**
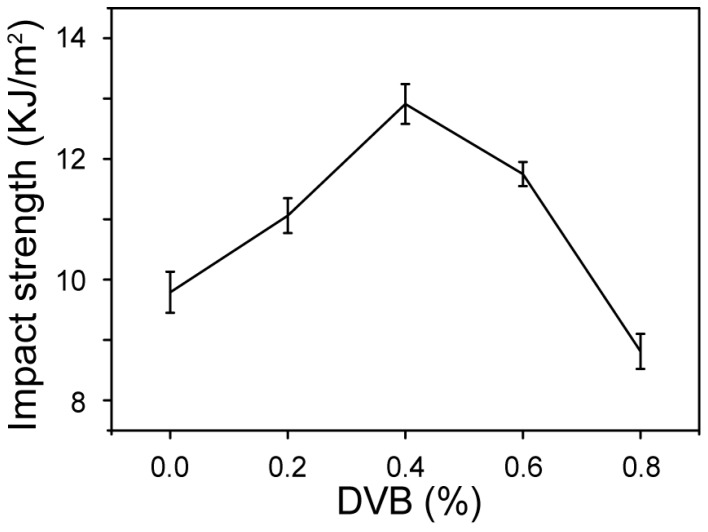
Changes in impact strength of WF/PP composites at various DVB concentrations.

Interestingly, when the DVB concentration increased from 0 to 0.4%, the composites exhibited a maximum improvement of 32% in impact strength; with a further increase in DVB concentration, the impact strength decreased (Figure 7). The marked improvement in impact strength could be largely associated with the strengthening of interfacial bonding between the modified matrix and WF, allowing the absorption of more energy during impact fracture [9]. At DVB concentrations higher than 0.4%, the melt flow rate of the matrix decreased and was accompanied by a higher degree of branching and crosslinking. This occurrence led to insufficient wetting of WF by the modified matrix, resulting in a weak fiber–matrix interface and inferior impact strength [30]. Under a similar MAH grafting level of MPPD0.4 and MPPD0.8 (Figure 3), the composites of WMPD0.4 exhibited superior mechanical properties owing to a higher melt flow rate in MPPD0.4 relative to that of WMPD0.8. The mechanical properties of WMPD0.8 were even lower than those of the ternary systems (WPMA), which could be attributed to the weak fiber–matrix interface. Moreover, this result indicated that the molecular structure of the modified matrix (such as the grafted polar monomer, molecular weight, and the branching and crosslinking structure) affected the wetting of WF by the matrix and the weak fiber–matrix interface.

#### 3.2.2. Dynamic Mechanical Analysis

**Figure 8:**
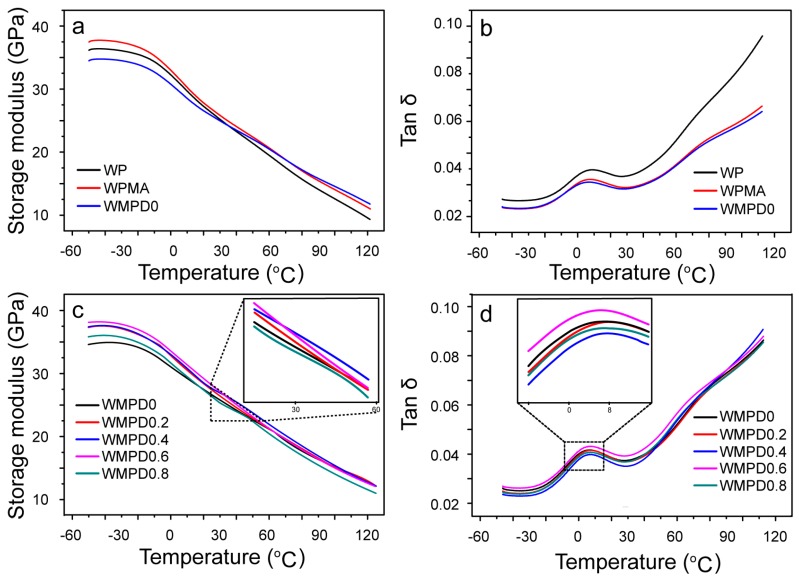
Dynamical mechanical properties: (**a**) storage modulus and (**b**) tanδ values of WP, WPMA, WMPD0, and WMPD0.4; (**c**) storage modulus; and (**d**) tanδ values of the composites modified with various DVB concentrations.

The variation in storage modulus (G′) and tanδ (damping factor) with temperature for different compositions of the composites is shown in Figure 8. Compared with G′ value of WP at temperatures ranging from −50 to 30 °C (Figure 8a), WPMA exhibited a higher G′ value, and WMPD0 obtained a lower G′ value. With a further increase in temperature above 80 °C, WMPD0 exhibited a higher value of G′ than that of WPMA. This result suggested that effective coupling occurred between WF and matrix in WMPD0 [30,31]. Figure 8b presents the corresponding tanδ curve of the unmodified and modified composites. Compared with the unmodified composites, the WPMA and WMPD0 composites showed a significant decrease in the damping factor. The decrease can be attributed to the effective coupling between WF and the matrix, which tends to dissipate less viscous energy, thereby obtaining a lower value for tanδ [32]. Moreover, WMPD0 exhibited a lower tanδ, compared with WPMA. The reason was that in higher WF content (60% in weight), the modified matrix underwent severe chain scission, which showed a tendency to improve WF dispersion in the composites, and strengthened the interfacial coupling. The immobilized PP matrices surrounding the fibers in the strong fiber–matrix interface hindered the matrix chain motion during relaxation, and then reduced the deformation or friction between the fibers and the matrix, thereby decreasing tanδ [33]. This trend was consistent with the aforementioned mechanical properties (Table 3).

Figure 8c,d show the effects of matrix modification with different DVB concentrations on the G′ and tanδ of the resulting composites. The G′ values of the composites were markedly increased by increasing the DVB concentration (Figure 8c). This increase in storage modulus may be attributed to the improved interfacial adhesion between WF and the modified matrix, as well as the decrease in degradation via the chain scission reactions of the modified matrix. However, with a further increase in the DVB concentration to 0.8% at a temperature above 30 °C, the lowest G′ value was observed. This trend was consistent with the aforementioned mechanical properties of the resulting composites (Figure 6 and Figure 7). This result indicated the critical concentration of DVB, beyond which the wetting ability of WF by the modified matrix and interfacial adhesion was decreased [34,35,36]. The damping factor, tanδ, was independent of the stiffness of the composites. The viscoelastic response of the composites was related to the nature of the individual composites, their fractions in the composites, and the fiber–matrix interface [31,35]. When WF content was constant, and the DVB concentration was increased to 0.4%, the damping factor of the modified composites decreased, which could be attributed to the restriction of chain mobility due to strengthened interfacial adhesion (Figure 8d). This increased adhesion, reduced the viscoelastic lag between the stress and strain, and less viscous energy dissipated, thereby decreasing tanδ. With a further increase in DVB concentration, the MAH grafting level decreased, and was accompanied by a higher degree of branching and crosslinking (Figure 4), indicating that matrix chain motion in WMPD0.8 is more difficult than that in WMPD0.6 [33,34]. Therefore, less viscous energy dissipated in WMPD0.8, and tanδ was lower in WMPD0.8 than in WMPD0.6. A higher melt flow rate and a similar MAH grafting level in MPPD0.4 (Figure 4) resulted in a stronger fiber–matrix interface than that in MPPD0.8; therefore, less viscous energy dissipated, and a lower tanδ was obtained in WMPD0.4 than in WMPD0.8.

#### 3.2.3. Fracture Morphology Analysis

**Figure 9:**
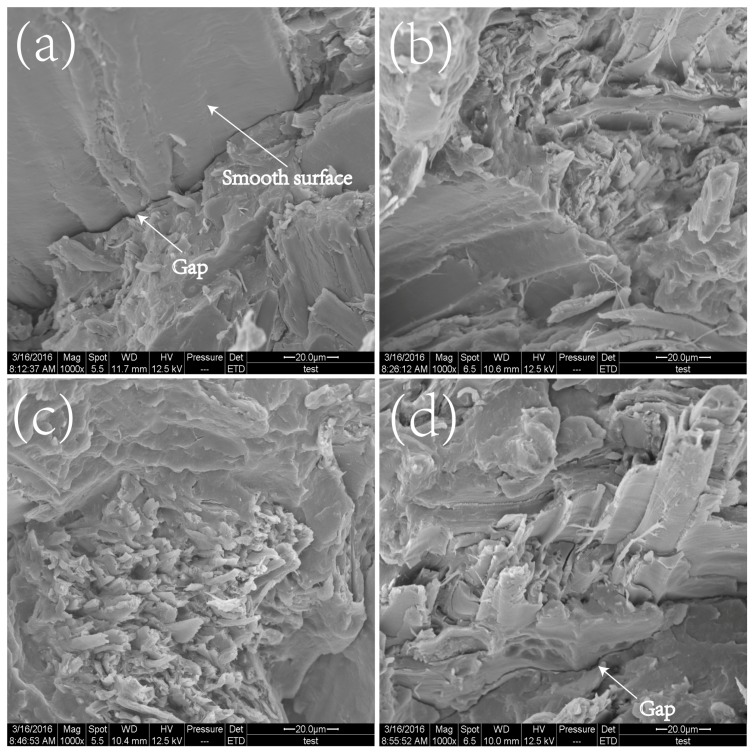
SEM macrographs of fracture surface of the composites. (**a**) WF; (**b**) WMPD0; (**c**) WMPD0.4; (**d**) WMPD0.8.

The fracture morphology of unmodified and modified composites was analyzed by SEM to provide an insight into fiber–matrix interactions (Figure 9). In the unmodified composites, WF with a clean and smooth surface was devoid of the PP matrices and an interfacial separation (Figure 9a). This occurrence indicated that a weak interface was formed between WF and the matrix [32]. In the composites based on the modified PP matrix, broken fibers were embedded in the modified matrix, and the interfacial boundary became indistinct, suggesting that the main energy dissipation of the applied stress was borne by the fracture of WF itself, other than debonding (Figure 9b,c). This occurrence could be attributed to the improved interfacial compatibility between WF and the modified matrix, as confirmed by the mechanical properties [9]. However, a coarse fracture surface was observed, when DVB concentration was added with a dosage of 0.8%. In addition, noticeable gaps at the interfaces were observed, suggesting a weak fiber–matrix interface (Figure 9d). As indicated from aforementioned mechanical data and DMA curves, the lower MFI values and excessive entanglement structure in MPPD0.8 resulted in a marked increase in melt viscosity at a high percentage of WF, which would result in the insufficiency of WF by the matrix, thereby forming a weaker fiber–matrix interface than that of WMPD0.4. Therefore, the stress from the matrix could not be transferred sufficiently to WF at the weak fiber–matrix interface. This finding provides evidence for the lower mechanical properties obtained in WMPD0.8, compared with that in WMPD0.4.

## 4. Conclusions

PP matrix modified by grafting with both MAH and DVB was compounded with wood fibers (60% in weight) to improve the interfacial compatibility of the resulting WF–PP composites. FTIR and torque analysis indicated that MAH and DVB were successfully grafted on the PP chain. The results demonstrated that the incorporation of DVB in PP–MAH grafting systems could improve the MAH grafting level, and partly offset matrix degradation as reflected by the improvement of tensile strength, thus improving the mechanical properties of the resulting composites. Compared with the traditional system containing the MAPP coupling agent, the composites based on the matrix modification with both MAH and DVB exhibited higher mechanical properties, particularly for impact strength (maximum improvement in nearly 32%). Dynamic mechanical analysis and fracture morphology analysis were in good agreement with the aforementioned mechanical results.

## Figures and Tables

**Table 1:** Formulations of modified polypropylene (PP) matrix in extruding.

Sample	Virgin PP	DCP (wt %)	MAH (wt %)	DVB (wt %)
MPPD0	100	0.09	0.4	0.0
MPPD0.2	100	0.09	0.4	0.2
MPPD0.4	100	0.09	0.4	0.4
MPPD0.6	100	0.09	0.4	0.6
MPPD0.8	100	0.09	0.4	0.8

The formulations of maleic anhydride (MAH), divinyl benzene (DVB), and dicumyl peroxides (DCP) are expressed by weight percentages based on Virgin PP.

**Table 2:** Formulations of composites in extruding.

Sample Type	WF Load (wt %)	Virgin PP (wt %)	Grafted PP (wt %)	MAPP (wt %)
WF/PP (WP)	60	40	-	-
WF/PP/MAPP (WPMA)	60	36	-	4
WF/MPPD0 (WMPD0)	60	-	40	-
WF/MPPD0.2 (WMPD0.2)	60	-	40	-
WF/MPPD0.4 (WMPD0.4)	60	-	40	-
WF/MPPD0.6 (WMPD0.6)	60	-	40	-
WF/MPPD0.8 (WMPD0.8)	60	-	40	-

The formulations of wood fibers (WF), PP, and maleated PP (MAPP) are expressed by weight percentages.

**Table 3:** Mechanical properties of WP, WPMA, and WMPD0.

Sample Type	Tensile Strength (MPa)	Tensile Modulus (GPa)	Flexure Strength (MPa)	Flexure Modulus (GPa)	Impact Strength (KJ/m^2^)
WP	16.0 ± 0.7	2.3 ± 0.1	26.5 ± 0.7	6.3 ± 0.1	5.6 ± 0.8
WPMA	42.5 ± 0.9	2.4 ± 0.1	73.7 ± 1.1	6.7 ± 0.1	9.0 ± 0.5
WMPD0	51.4 ± 0.6	2.1 ± 0.2	81.6 ± 0.9	5.6 ± 0.2	9.8 ± 0.6
